# Editorial: Electroencephalography and other neuroelectrophysiologic studies in post-MRI generation veterinary medicine

**DOI:** 10.3389/fvets.2024.1396967

**Published:** 2024-04-09

**Authors:** Fiona M. K. James, Marcin Wrzosek, Daisuke Hasegawa

**Affiliations:** ^1^Department of Clinical Studies, Ontario Veterinary College, University of Guelph, Guelph, ON, Canada; ^2^Department of Internal Medicine and Clinic of Diseases of Horses, Dogs and Cats, Wrocław University of Environmental and Life Sciences, ul., Wrocław, Poland; ^3^NeuroTeam Specialist Veterinary Clinic, Wrocław, Poland; ^4^Vet Imaging Veterinary Computed Tomography, Kraków, Poland; ^5^Department of Veterinary Medicine, Nippon Veterinary and Life Science University, Musashino, Japan

**Keywords:** electroencephalography—diagnosis, electroencephalography—methods, electrical impedance myography (EIM), somatosensory evoked potentials (SEPs), veterinary

Neuroelectrophysiologic studies have long been integral for functional evaluations of both the central and peripheral nervous systems. In the era preceding the widespread use of magnetic resonance imaging (MRI) and computed tomography (CT) scans, these tests played a crucial role. However, the advent of advanced imaging, particularly MRI, shifted the paradigm in veterinary medicine, seemingly eclipsing the importance of neuroelectrodiagnostics. The assumption was that imaging could provide a comprehensive understanding of both structure and function.

This Research Topic explores the evolving landscape of neuroelectrophysiologic studies in the post-MRI era. Despite the popularity of advanced imaging techniques, veterinarians are re-evaluating the scope and significance of neuroelectrodiagnostics. It is increasingly evident that diagnostic imaging, while proficient in detecting structural abnormalities, falls short in documenting crucial functional aspects of the nervous system.

Several facets of electroencephalography (EEG) are explored in this Research Topic. The first theme comprises advancements in EEG techniques. Luca et al. presented a comprehensive survey of EEG usage and techniques in veterinary neurology. Findings revealed underutilization and barriers such as equipment availability, highlighting the need for standardization and harmonization of EEG techniques in canine neurology. In response, to propose routine clinical short-term video-electroencephalography (vEEG) recordings, Lyon et al. demonstrated its feasibility in unsedated dogs and cats. This standardized, unanesthetized procedure proved clinically informative, positioning EEG as a vital first-line neurological functional exploration test. Folkard et al. ventured into homes, conducting in-home vEEG and actigraphy recordings. Their approach, integrating vEEG and actigraphy, not only assesses behavior but also detects epileptic seizures. This innovative study laid the groundwork for holistic insights into canine behavior in natural settings.

A second theme involved the use of EEG, to understand for example pharmaceutical effects or age- or sleep-related features. Mizuno et al. explored ketamine in cats with temporal lobe epilepsy. Beyond sedation, ketamine emerged as an inducer of epileptiform discharges during EEG recordings. This study prompts a reconsideration of sedation alternatives, offering a nuanced perspective on the potential activating effects of pharmacologic choices on neuroelectrophysiologic studies. Simultaneously, Pellegrino and Gómez Álvarez employed xylazine sedation to unravel EEG features of the developing canine brain. Their meticulous analysis provides a refined understanding of EEG characteristics, emphasizing age-specific interpretation in veterinary neuroelectrophysiology. Examining the senescent brain, Mondino et al. scrutinized sleep-wakefulness cycles and EEG features in senior dogs. Their polysomnographic study unveiled age-related changes correlated with cognitive performance, offering potential markers for canine cognitive dysfunction syndrome.

Williams et al. extended the scope of EEG to the marine world, examining EEG in stranded California sea lions. Abnormalities in EEG recordings shed light on domoic acid toxicosis, emphasizing the importance of understanding EEG patterns in marine mammals. Knipe et al. took a visual perspective, focusing on periodic discharges in veterinary EEG. Their review explores these enigmatic patterns, challenging our understanding and paving the way for further research into the interpretation of these patterns in clinical practice.

Extending neurophysiology to muscle health and neurosurgery was the final theme in this post-MRI compilation. Verga et al. delves into the realm of canine skeletal muscle health, presenting normative values and repeatability for electrical impedance myography. Their study provides a convenient and non-invasive tool for assessing muscle condition, offering insights into age-related changes and gender differences. Okuno et al. address a critical aspect of neurosurgery, evaluating the impact on spinal cord integrity during thoracolumbar intervertebral disk herniation surgery in dogs. Utilizing somatosensory-evoked potentials, they provide a framework for maintaining electrophysiological safety during surgical interventions.

This Research Topic of articles not only establishes the current state of knowledge in post-MRI veterinary neuroelectrophysiology but also propels the field forward with innovative methodologies and insights. These contributions establish a foundation for the future, where neuroelectrophysiology intertwines seamlessly with veterinary clinical practice. Given the rapid development of diagnostic technologies for neurological diseases in animals, both advanced imaging diagnostics (CT and MRI) and all electrodiagnostic techniques, their fusion may be predicted, with consequential development of hybrid diagnostic solutions in veterinary medicine.

## Author contributions

FJ: Conceptualization, Writing – original draft, Writing – review & editing. MW: Conceptualization, Writing – review & editing. DH: Conceptualization, Writing – review & editing.

